# Aflatoxin exposure among children of age 12–59 Months in Butajira District, South-Central Ethiopia: a community based cross-sectional study

**DOI:** 10.1186/s12887-022-03389-w

**Published:** 2022-06-02

**Authors:** Mary Ayele, Demewoz Haile, Silvia Alonso, Heven Sime, Adugna Abera, Kifle Habte Balcha, Kedir Teji Roba, Geremew Tasew Guma, Bilal Shikur Endris

**Affiliations:** 1 Doctors With Africa CUAMM, Medici Con L’Africa, Cuamm, Ethiopia; 2 Department of Nutrition and Dietetics, School of Public Health, Addis Ababa University, Addis Ababa, Ethiopia; 3 Animal and Human Health Program, International Livestock Research Institute, Addis Ababa, Ethiopia; 4 Bacterial, Parasitic and Zoonotic Diseases Research Directorate, Ethiopian Public Health Institute, Addis Ababa, Ethiopia; 5 Food Science and Nutrition Research Directorate, Ethiopian Public Health Institute, Addis Ababa, Ethiopia; 6 College of Health and Medical Sciences, Haramaya University, Dire Dawa, Ethiopia

**Keywords:** Aflatoxin, Children, Butajira, Ethiopia, Cross-sectional

## Abstract

**Background:**

The continued provision of safe food, free of aflatoxin remains a huge challenge in developing countries. Despite several favourable climatic conditions that facilitate aflatoxin contamination in Ethiopia, there is little information showing aflatoxin exposure in children. Therefore, this study assessed aflatoxin exposure among young children in Butajira district, South-Central Ethiopia.

**Methods:**

Community based cross-sectional study stratified by agro-ecology was employed in Health and Demographic Surveillance Site (HDSS) of Butajira. The study included 332 children aged 12–59 months and were selected by simple random sampling technique using the HDSS registration number as a sampling frame. We collected data on dietary practice and aflatoxin exposure. Aflatoxin M1 concentration in urine was measured by Enzyme-Linked Immunosorbent assay (ELISA). The data analysis was carried out using STATA.

**Results:**

Detectable urinary Aflatoxin M1 was found in 62.4% (95% CI: 56.9 – 67.5%) of the children at a level ranging from 0.15 to 0.4 ng/ml. Children living in lowland agro-ecological zone had [AOR = 2.11 (95% CI; 1.15, 3.88] odds of being exposed to aflatoxin as compared to children living in highland agro-ecological zone. Children at lower socio-economic status [AOR = 0.27 (95% CI; 0.14, 0.50] and medium socio-economic status [AOR = 0.47 (95% CI; 0.25, 0.87] had 73% and 53% lower odds of being exposed to aflatoxin as compared to children in the higher socio-economic status, respectively.

**Conclusions:**

Aflatoxin exposure among young children was very high in South-Central Ethiopia. This high aflatoxin exposure might emphasize the need for aflatoxin exposure mitigation strategies in Ethiopia. Especially, raising awareness of the community towards aflatoxin exposure is very crucial. In addition, further research is required to assess long-term aflatoxin exposure and its association with child growth and development.

## Background

Aflatoxins are one family of mycotoxins, and are a naturally occurring toxic by-product, named after the genus of fungus that produces it (*Aspergillus flavus and Aspergillus parasiticus*) [1]. Aflatoxins are largely associated with agriculture commodities produced in the tropics & subtropics [2]. Aflatoxin may enter the food supply by direct contamination of food products resulting from mould growth on food, or by indirect contamination through the use of contaminated ingredients in processed food or through use of animal products such as milk, milk products, eggs or meat [3]. Pre- and post-harvest crop management has a significant influence on the accumulation of aflatoxin in dietary staples [4]; thus populations highly reliant on these staples and with limited agricultural capacity and storage facilities are most frequently exposed through diets [5]. The use of aflatoxin metabolites as biomarkers has been a common approach to understand individuals’ dietary exposure to these toxins, but also uptake, toxicokinetics and toxicodynamics of these toxins [6]. Aflatoxin M1 (AFM1) has been well established as a biomarker of exposure for the recent (24-72 hours) ingestion of Aflatoxin B1 (AFB1) and is the most frequently detected urinary aflatoxin [7].

In many parts of the developing world, exposure to aflatoxins at high levels remains a significant health burden [8]. It is estimated that approximately 4.5 billion people, predominantly those living in developing countries, are at risk of exposure to aflatoxins [5]. A study done in Northern Ethiopia by A. Ayelign et al (2016) among children aged 1 to 4 years, reported that aflatoxin M1 was found in 7% out of 200 urine samples analysed with a mean concentration of 0.064ng/ml [9]. According to Partnership for Aflatoxin Control in Africa (PACA) report in 2014, children under-five remain particularly vulnerable to aflatoxin exposure significantly hindering children’s growth and development while damaging their immunity [10]. In Ethiopia, the prevalence of impaired growth is still worryingly high, where the proportion of stunting in children under 5 years is 38% [11]. Moreover, animal studies provide evidence that chronic aflatoxin exposure retards growth and interferes with micronutrient absorption and utilization [12].

Many of Ethiopia’s ecosystems are among the most favourable for aflatoxicogenic fungi and aflatoxin contamination [13]. Features conducive of a possible high contamination of food and feed products in Ethiopia include its climatic conditions, traditional crop production practices, inadequate harvesting, drying and storage practices, limited policy and institutional capacity in assessment and management of fungal contamination in agricultural products, lack of awareness and high reliance on one or two primary crops constituting the main component of the diet [13]. Likely as a result of this, major staple grain crops in the country have been reported to be contaminated with aflatoxin [14–16]. Despite this, there is limited evidence on the levels of aflatoxin exposure in children. Considering the presence of several favourable conditions that facilitate aflatoxin contamination of foods and the high prevalence of impaired child growth in South-Central Ethiopia, this study assessed aflatoxin exposure among children aged 12 to 59 months in Butajira District, Southern Ethiopia. Additionally, we assessed the type of food consumed by the children.

## Materials and methods

### Study setting and sampling procedure

**Fig. 1:**
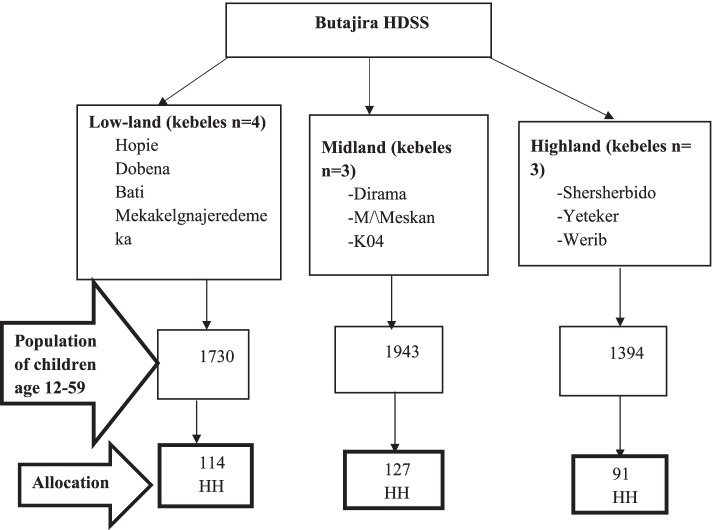
Sampling procedure of children in Butajira HDSS, 2018

The study was conducted in Butajira Health and Demographic Surveillance Site (HDSS), which is located l30 km south of Addis Ababa. The HDSS contains 10 kebeles spread through three agro-ecological zones; Highland, Midland and Lowland. Three of the kebeles; Shersherbido, Yeteker and Werib are located in the Highland agro-ecological zone. Dirama, Misrak Meskan & K04 kebeles are found in the Midland agro-ecological zone and the rest 4 kebeles; Hopie, Dobena, Bati & Mekaklegna jeredemeka are found in the Lowland agro-ecological zone (fig. 1). There is difference in altitude, temperature and precipitation level among the three agro-ecological zones [17]. The HDSS estimated total population in 2018 was 80,369 (taken from HDSS database), from which children 12-59 months of age accounted for 6.3%. Enset (False banana), Teff, maize, millet, barley and legumes are the staple foods in the area [17].

We employed a cross-sectional study, with stratified sampling based on agro-ecological zone. First, we allocated the sample size proportional to the number of children in each agro-ecological zone using the HDSS registration number as a sampling frame. Second, in order to include all the kebeles within each agro-ecological zone, we allocated the sample size proportional to the number of children in each kebele. Finally, we applied a simple random sampling technique to select the households in each kebele. The data collectors used the following steps to select the allocated sample from each kebele: 1) went to the point in the kebele where the population was about equally distributed on all sides; 2) selected a smooth and level spot where one can spin a ballpoint pen; 3) spin the pen; 4) the data collectors determined in which direction the ballpoint of the pen was pointing and went to that direction; 5) the first household in the kebele in that direction became the starting household. Then, households with the target children were consecutively selected until the desired sample size was archived. Only one child between 12 and 59 months living in the house for at least 6 months was recruited from each selected household; whenever more than one eligible child was found, lottery method was applied to select one of them.

### Sample size calculation

The sample size required to meet the objective of this study was 306. However this study was a part of another study (unpublished) which required a higher sample size (*n*=332). Thus the sample size (*n*=332) we used for this specific objective provided adequate power.

The sample size for the first objective is calculated using single population proportion formula based on the following assumptions: *P*= prevalence of Aflatoxin M1 in urine as 7%, from a study done in Ethiopia in 2017 [9].

- D (margin of error) = 3%
- 95% of confidence interval
- N = number of sample
- Sample size determination will be as follows
- N = Z2α/2 p (1-p)/d2
- N = 278

Sample size for the second objective is calculated using two population proportion formula in open epi software, based on the following assumption:

- P1 = prevalence of Stunting as 52.5%, from a study done in Butajira in 2017 [18]
- Zα/2 = standard score corresponding 95% confidence interval (1.96)
- Zβ = standard score corresponding 80% power (0.84)
- Odds ratio = 2
- r = ratio between group one & group two as 1
- By using the following assumption, the sample size will be 151 in each group, making a total sample size of 302
- Since, sample size for the second objective is greater than the first, 302 is taken.
- Adding a contingency of 10% for non-respondent
- Final sample size is 332

### Data collection approach

We collected data on socio-demographic characteristics of the households and dietary practices. Data was collected by a team of trained data collectors using structured and pre-tested questionnaires. The questionnaires were translated into Amharic and retranslated back to English to check and maintain its consistency. The data was collected in paper by using Amharic version of the questionnaire.

### Dietary assessment of the index child

A Food frequency questionnaire (FFQ) was used to identify the types of foods consumed in the three days (72hours) previous to the day of the visit. The questionnaire collected information on the frequency of consumption (i.e. number of times per day, and per week) and the usual portion size consumed in grams. The food items listed in the FFQ were food items known to be consumed in the study area and known to be vulnerable to aflatoxin contamination based on previous studies in Ethiopia [10, 14, 16].

### Urine sample collection and handling

Random urine samples were collected on the day of the visit using a 10ml urine cup by the help of the mother/care giver of the index child. The cup was labelled immediately after urine collection using a sticker with a unique identifier code, and subsequently recorded in the questionnaire. Urine samples were placed in portable freezers. At the end of each data collection day, the collected urine samples were checked for consistency by the field coordinator, then transferred in to 10ml freezing tubes and kept at Butajira Health Center at -20 C^0^ until analysis. After the end of the data collection period, all the collected urine samples were transported to Ethiopian Public Health Institute (EPHI) for laboratory analysis.

### Laboratory analysis

#### Urine sample and reagent preparation

For the determination of urinary concentration of AFM1, a competitive ELISA kit (from Helica Biosystems Inc.Cat.No.991AFLM01U-96) was used and procedures were based on the protocol obtained from the kit manufacturer [19]. Prior to lab analysis, urine samples and reagents were brought at room temperature. Five millilitres of urine sample were aliquated into centrifuge tubes and centrifuged at 3000rmp for 10 minutes. Phosphate Buffer Saline-Tween packet (PBS with 0.05% Tween20) was reconstituted by washing out the contents with distilled water into 1 litter glass. The PBS was stored refrigerated (2-8C^0^) when not in use. Standard optimization was carried out before working with the samples, in order to get the same readings in the standards value as stated in the protocol.

#### Assay procedure for determination of aflatoxin M1 in urine using Enzyme Linked Immunosorbent Assay (ELISA)

Nine hundred and fifty microliters (950) of distilled water were pippeted into a 1.5ml micro tube, then each 50µl of standards and urine supernatant were added into the 950µl of distilled water in the tube to make-up a total of 1000µl. It was mixed by vortex mixer for 10 second. Two hundred microliters of the assay-buffer were added into the mixing well, then a 100µl of the diluted standards containing aflatoxin M1 ranging from 0 to 4000 ppt (0.0-4.0ng/ml) and urine samples were added into the mixing well, to make-up 300µl. These were mixed by priming pipette at least 5 times. By using new pipette, a 100µl of the mixture were transferred into the antibody coated micro-wells in duplicate and incubated for 1hr at room temperature. The contents of the antibody coated micro-wells was decanted in to discard basin, then each micro-wells were filled with PBS-Tween packet and decanted into a discard basin for 3 times. A hundred microliters of conjugate were then added into each micro-well and incubated at ambient temperature for 15 minutes. The plate was washed and a hundred microliters of substrate reagent (Tetramethylbenzidine) was added into each well and incubated at room temperature for 15 minutes in the dark. A hundred microliters of stop solution were added into each of the wells using a multichannel pipette. The intensity of the solution colour in the micro-plate was measured optically using an ELISA reader (Bio-Rad) with an absorbance filter of 450nm as soon as stop solution was added. The optical densities (OD’s) of the samples were compared to the OD’s of the standards and an interpretative result was determined. Aflatoxin concentration is indirectly proportional to the optical density [19].

### Data analysis

The data were entered in to Epi-data version 4.0 for windows, and then exported to STATA version 14 for analysis.

Principal Component Analysis (PCA) was used to construct the wealth index to classify the households into low, medium and high socio-economic status in STATA using the socio-economic data about the households. All the variables included in PCA were those we thought are appropriate to explain the wealth of the households in the study area. ‘Rule of thumb’ was used to select the variables used to run PCA, where variables with prevalence below 5% or above 95% were excluded from the analysis. Ten variables were included in PCA. The questions for the variables were as follows: ‘house ownership’, options 1= private, 2= government, 3= rent, 4= relatives/others house, 5= other (specify)? We coded ‘other’ into the appropriate categories. Dichotomous response variable was created; 1(yes) = private and 0(no) = 2, 3, & 4; ‘Material used for roof construction’, recorded 1= corrugated iron or metal and 0= Thatch/leaf/mud/plastic/bamboo/wood planks. ‘Other’ were coded into the appropriate categories; ‘Does your household has a functioning radio?’ 1= yes and 0= no; ‘Does your household use solar energy?’ 1= yes and 0= no; ‘Does any member of this household own a mobile phone?’ 1= yes and 0= no; ‘How many rooms does your house have?’ 1= three & more rooms and 0= less than three rooms; ‘What kind of toilet facility do members of your household usually use?’ 1= flush or pour flush/pit latrine with slab and 0= pit latrine without slab/no facility/bush/field, ‘Other’ were coded into the appropriate categories; ‘what is the main source of drinking water for members of your household?’ 1= piped inside dwelling or yard/public tab/protected well/protected spring and 0= unprotected well/pound/lake/river/stream/dam; ‘Does this household own any livestock, herds, other farm animals or poultry?’ 1= yes and 0= no; ‘Do you have separate room used as kitchen?’ 1= yes and 0= no.

PCA was run with all the above ten variables and the first component (component one) was taken to represent the household’s wealth as it accounts for the largest proportion of the variance. The first, second and third eigenvalues were 2.37, 1.74 and 1.34 respectively. The Kaiser-Meyer-Oiklin value was 0.65, exceeding the recommendation value of 0.6 and the Bartlett’s test of sphericity reached statistical significance (p<0.001). Assuming socio-economic status (SES) to be uniformly distributed, the households were divided in to three quintiles; low, medium and high socio-economic status based on the wealth index score by using STATA. Table 1 below shows the mean wealth index score by quintile.

**Table 1:** Mean wealth index score by quintile in Butajira District, 2018

Quintile	Frequency (*n* = 332)	Mean
Low SES	111	-1.89
Middle SES	117	0.42
High SES	104	1.54

Descriptive statistics such as mean, standard deviation and frequency were used to summarize household characteristics. We employed univariable logistic regression model to identify potential candidate variables to be included in the multivariable logistic regression model. Variables with p-value < 0.25 in the univariable model [20] were included in the multivariable logistic regression model. We calculated odd ratios with their 95% CI and p-value <0.05 in the multivariable logistic regression model determined as a level of significance.

## Results

### Socio-demographic characteristics of participants

A total of 332 children aged 12 to 59 months were included in the study, of which 98.5% (327/332) provided a urine sample. The mean age of the children in the study was 39 months (SD ± 10.9 months). Distribution of the study subjects by socio demographic and economic characteristics is presented in Table 2. The data on maternal characteristics showed that, 78% were housewives and 51% of them didn’t attend any formal education.

**Table 2:** Socio-demographic and economic characteristics of children aged 12–59 months in Butajira District, South-Central Ethiopia, 2018

**Characteristics**	N	Percent (%)
**Agro-ecology zone**		
Highland	93	28.0
Midland	127	38.3
Lowland	112	33.7
**Child age in month**:		
12–23 months	25	7.5
24–35 months	81	24.4
36–47 months	140	42.2
48–59 months	86	25.9
**Sex of the child**:		
Male	210	63.0
Female	122	36.8
**Maternal occupation**:		
Employed *(government/private employee, merchant, daily labourer & farmer)*	63	22.0
Not employed/*Housewife*	269	78.0
**Maternal educational Status**:		
No formal education	170	51.0
Primary education	137	41.0
Secondary or higher education	25	8.0
**Household socio-economic status (SES) categories:**		
Low SES	111	33.4
Medium SES	117	35.2
High SES	104	31.3

Total children (*N*) = 332

### Food intake by the children

Based on the three days’ recall, the children consumed maize (in the forms of ‘Kita’; a flat bread) (78.6%), Teff (in the forms of ‘Enjera’; a pancake) (26.1%), wheat (in the forms of bread) (78.3%) and broad bean (in the form of ‘Shiro Wot’; a stew) (39.3%). In addition, 23.5% of the children had cow milk at least once in the preceding three days (Table 3).

**Table 3:** The proportion of children consumed the food product at least once in the past 72 h across agro-ecological zone in Butajira District, 2018

Food category	Agro-ecological zones			Total (*N* = 332)
	Highland (*n* = 93)	Midland (*n* = 127)	Lowland (*n* = 112)	
**‘Kita’**- flat bread, made of:				
Maize	84 (90.3%)	78 (61.4%)	99 (88.4%)	261 (78.6%)
Maize & wheat blend	0	0	1 (0.9%)	1 (0.3%)
Didn’t eat ‘Kita’ within 72 h	9 (9.7%)	49 (38.6%)	12 (10.7%)	70 (21.1%)
**‘Enjera’**- a pancake made of:				
Teff	4 (4.3%)	37 (29.0%)	8 (7.1%)	49 (14.8%)
Maize	25(26.9%)	3 (2.4%)	30 (26.8%)	58 (17.5%)
Teff & maize blend	20(21.5%)	26 (20.5%)	30 (26.8%)	76 (22.9%)
Sorghum & Teff blend	0 (0%)	2 (1.6%)	1 (0.9%)	3 (0.9%)
Sorghum & maize blend	0 (0%)	0 (0%)	2 (1.8%)	2 (0.6%)
Didn’t eat ‘Enjera’ within 72 h	44 (47.3%)	59 (46.5%)	41(36.6%)	144 (43.3%)
**Bread**- made of:				
Wheat	32 (34.4%)	66 (52.0%)	32 (28.6%)	130 (39.2%)
Wheat & maize blend	4 (4.3%)	17 (13.4%)	7 (6.2%)	28 (8.4%)
Sorghum & maize blend	1 (1.1%)	5 (3.9%)	2 (1.8%)	8 (2.4%)
Didn’t eat bread within 72 h	56 (60.2%)	39 (30.7%)	71 (63.4%)	166 (50%)
**Stew ‘Shiro wot’**- made of:				
Broad bean	21 (22.6%)	14 (11.0%)	13 (11.6%)	48 (14.5%)
Pea	10 (10.8%)	14 (11.0%)	3 (2.7%)	27 (8.1%)
Pea, chicken pea, broad bean & cassava blend	1 (1.0%)	16 (12.6%)	6 (5.4%)	23 (6.9%)
Pea, chicken pea & broad bean blend	6 (6.5%)	11 (8.7%)	5 (4.5%)	22 (6.6%)
Pea & chicken pea blend	0 (0%)	2 (1.6%)	0 (0%)	2 (0.6%)
Didn’t eat ‘Shiro wot’ within 72 h	55 (59.1%)	70 (55.1%)	85 (75.8%)	210 (63.3%)
**Cow milk**	16 (17.2%)	44 (34.6%)	18 (16.1%)	78 (23.5%)
Didn’t have cow milk	77 (82.8%)	83 (65.4%)	94 (83.9%)	254 (76.5%)

Maize was relatively consumed higher in the Lowland ago-ecological zone in the form of ‘Kita’ (37.9%) and ‘Enjera’ (51.7%). Teff, wheat, pea and cow milk were mainly consumed in the Midland agro-ecological zones (Table 3).

Among the studied households, maize was the most commonly (85.5%) crop stored during the data collection period followed by Teff (13%). In addition, 61% of the households used silos to store their crops and majority (69.7%) of them stored the crops for more than five weeks (Table 4).

**Table 4:** The type of stored crop, storage material and duration of crop storage among the studied households in Butajira District, 2018

Category	Frequency (*n* = 332)	Percent (%)
**Type of stored crop in the household**:		
Maize	284	85.5
Teff	43	13.0
Wheat	4	1.2
Sorghum	1	0.3
**Type of storage material**:		
Silos	203	61.1
Plastic bugs	95	28.6
‘Gotera’, made from wool or bamboo	23	7.0
‘Gota’, made from Teff, straw & mud	11	3.3
**Duration of crop storage**:		
More than three months	5	1.5
Between three month to five weeks	225	67.8
Between five to two weeks	90	27.1
One week maximum	12	3.6

### Aflatoxin exposure among the study participants

Three-hundred-twenty-seven (327) urine samples from children aged 12-59 month were tested for Aflatoxin M1(AFM1) out of which, detectable urinary AFM1 was found in 62.4% (95% CI; 56.9, 67.5%). Samples with detectable urinary AFM1 ranged from 0.15ng/ml to 0.4ng/ml.

### Factors associated with aflatoxin exposure

In the univariable logistic regression model; except sex of the child all the variables in the model meet the significance level (p-value <0.25) to be included in the multivariable logistic regression model.

In the final multivariable logistic regression model, agro-ecological zone and socio-economic status were associated with aflatoxin exposure (p-value <0.05). Children living in lowland agro-ecological zone had [AOR= 2.11 (95% CI; 1.15, 3.88] odds of being exposed to aflatoxin as compared to children living in highland agro-ecology zone.

Children at lower socio-economic status had [AOR= 0.27 (95% CI; 0.14, 0.50] 73% lower odds of being exposed to aflatoxin as compared to children in the higher socio-economic status. Those children from medium socio-economic status had [AOR= 0.47 (95% CI; 0.25, 0.87] 53% lower odds of being exposed to aflatoxin than children in the higher socio-economic status (Table 5).

**Table 5:** Results of Multivariable logistic regression model of children aged 12–59 month in Butajira District, South-Central Ethiopia, 2018

	Dependent Variable: Aflatoxin Exposure			
Independent Variables	Frequency	Crude OR (95% CI)	Adjusted OR (95% CI)	*P*-Value (AOR)
Agro-ecological zone				
Highland	92	1	1	
Midland	123	1.45 (0.84, 2.53)	1.62 (0.91, 2.89)	0.102
Lowland	112	1.77 (1.00, 3.14)	2.11 (1.15, 3.88)	0.016
Age				
12–23 Months	24	1	1	
24–35 Months	80	0.50 (0.18, 1.39)	0.57 (0.19, 1.72)	0.318
36–47 Months	138	0.49 (0.18, 1.31)	0.49 (0.17, 1.44)	0.198
48–59 Months	85	0.64 (0.23, 1.79)	0.75 (0.24, 2.27)	0.607
Socio-Economic Status (SES)				
High SES	102	1	1	
Medium SES	116	0.44 (0.24, 0.80)	0.47 (0.25, 0.87)	0.015
Low SES	109	0.29 (0.16, 0.54)	0.27 (0.14, 0.50)	0.000
Maternal educational status				
Secondary or Higher education	24	1	1	
Primary education	136	0.57 (0.22, 1.47)	0.79 (0.29, 2.15)	0.646
No formal education	167	0.75 (0.29, 1.92)	1.22 (0.45, 3.35)	0.696

## Discussion

This study assessed aflatoxin exposure among young children aged 12-59 months by detecting urinary Aflatoxin M1 using ELISA and found high (62.4%) prevalence of aflatoxin exposure, with detectable level ranging from 0.15 ng/ml to 0.4 ng/ml. The presence of urinary biomarkers of aflatoxin is indicative of acute exposure (i.e. exposure having occurred in the previous 72 hours) [8] ,so in this study almost two third of children had been exposed to aflatoxins in their diets. As there is no safe threshold for aflatoxin exposure any level of exposure is considered a risk [8].

Our study found higher prevalence and concentration of aflatoxin M1 level than a study done in Northern Ethiopia, were AFM1 was detected in 7% of the study participants with a range 0.064-0.0070 ng/ml [9]. Other studies done in Cameroon [21] and Nigeria [7] also reported a prevalence of 14% (range: 0.06-4.7ng/ml ) and 14.2% (mean: 0.3ng/ml; SD: 0.4) of AFM1 in urine respectively. The difference in prevalence of aflatoxin exposure between the above cross-sectional studies and this study could be attributed to two major factors. The studies used LC-MS/MS for detection of aflatoxin in urine, which has high specificity, but made the possibility for trace detection difficult [22]. In addition, the studies have relatively small sample size than this study, where sample size determines the power of detecting the magnitude of aflatoxin exposure. Seasonal difference could be one major reason for the difference in prevalence of aflatoxin exposure in our study and the other from Northern Ethiopia [9]. That particular study was conducted on January 2016 and reported that Teff was the main food item consumed by the study participants. In Ethiopia, Teff is grown in the ‘belg’ rainy season (July to October) and harvested on November & December [23]. Even though aflatoxin contamination could happen during pre- harvesting period, the children might consumed Teff stored for less than one month or not stored at all. In contrast, our data was collected on July 2018 and maize was the main food item consumed by the study participants. Maize is usually grown in ‘Maher’ rainy season (June to September) and harvested on October & November [23] so, the children might consumed stored maize for more than 6 months. The longer the storage time, the greater the possibility of building up environmental conditions conducive to aflatoxigenic mould proliferation and subsequent mycotoxin production [24].

Similar to our study, studies done in Tanzania [25], Kenya [26] and China [27] analysed the presence & level of AFM1 in urine using ELISA and found a prevalence of 86%, 79.2% and 84% respectively. On the other hand, studies done in Ghana in 2010 [28] and 2015 [29] using HPLC, detected AFM1 in 91.2% and 100% of the analysed urine samples of children respectively. All the above studies conducted using either ELISA or HPLC reported relatively higher prevalence of AFM1 in urine compared to our study. This could be attributed to various factors such as difference in age, level of aflatoxin contamination in food items consumed by the study participants or individual variation in the toxicokinetics of mycotoxins. In addition, we collected random urine samples; while most of the above studies collected first morning urine samples, which are more concentrated and might lead to high level of AFM1. A very sensitive detection system like HPLC and ELISA allow detection at very low pictogram/ml compared to LC-MS/MS [22]. This study showed that ‘Kita’: a flat bread made from maize, ‘Enjiera’: a pancake prepared from Teff & maize blend, bread prepared from wheat, ‘Shiro wot’: stew prepared from broad bean and cow milk were the mainly consumed food items in the three days dietary recall. Even though we didn’t analysed the presence and level of aflatoxin in the food items consumed, different studies done in Ethiopia showed that the food items consumed similar to the study participants were prone to contamination by aflatoxin. A survey done by Ayalew. A in 2010, aflatoxins were detected in 88% of maize samples with a concentration of 4.1µg/kg [15]. A survey by Alemu et al; found contamination of maize with aflatoxin B1 (AFB1) in Southern Ethiopia with concentration of 22.72µg/kg [14]. Furthermore, according to a report by USAID in 2011, aflatoxin B1 was detected in four major crops of Ethiopia: barley, sorghum, Teff and wheat [16].

Our study found a statistically significant association between urinary aflatoxin exposure and agro-ecological zone. Children living in lowland agro-ecological zone had about 2 folds higher odds of being exposed to aflatoxin as compared to children living in highland agro-ecological zone. This difference in aflatoxin exposure by agro-ecological zone might be attributed to the difference in the temperature, humidity and moisture level, which can influence the growth of toxigenic mould and aflatoxin production on food items. In Ethiopia lowlands have high temperature (20 C^0^ to 27 C^0^) compared to highlands (10 C^0^ to 16 C^0^) [30]. Aflatoxins can develop within 24 hours in mold and fungi infected maize stored under conditions of high moisture (above 14%) and higher temperatures (26.6 C^0^) [31]. Similarly, studies done in Tanzania [32], Nigeria [33] and Kenya [34] found a statistically significant difference in aflatoxin exposure across different agro-ecological zones.

Our study also found that, children in lower socio-economic status and medium SES had 73% and 53% lower odds of being exposed to aflatoxin as compared to children in the higher socio-economic status respectively. In contrast a study done in Ghana [35] reported that higher income was associated with 30%- 40% reduced odds of high aflatoxin-albumin adduct level. Similarly, study done in Kenya [36] reported that aflatoxin-albumin adduct in women living in the worst socioeconomic conditions were 4.7- 7.1 times higher than those with the best socio-economic status. This difference in results might be that households at higher socio-economic status consume more purchased commodities such as bread, peanut and milk in the market, which might be contaminated with aflatoxin. In subsistence farming households, those households with higher socio-economic status store foods for longer duration than low SES households. In some areas storing food for longer time is considered as an indicator of wealth. This might contribute for this difference; however, it is not precisely clear why we found higher odds of aflatoxin exposure among children from higher SES. There were also studies done in Benin [37] and Ghana [38], which didn’t found a statistically significant correlation between socio-economic status and aflatoxin exposure (p< 0.05).

The strength of this study can be seen in terms of using ELISA to analyse the level of AFM1 in urine, which is a highly sensitive analytical method, simple, rapid, preferred to analyse large samples and made trace detection possible as the excretion rate of aflatoxin M1 through kidneys is very low. However, ELISA has issues with specificity, where compounds with similar chemical groups as AFM1 can also interact with the antibodies. But, again this has been argued by Groopman et al, in that AFM1 is the most common metabolite of AFB1 in urine, so results are unlikely to be distorted [25]. While interpreting the results obtained from this study, recall bias in the case of food frequency questionnaire and the cross-sectional nature of the study need to be taken in to consideration.

## Conclusion

This study showed that the prevalence of aflatoxin exposure is high among children aged 12 to 59 months. Thus, aflatoxin exposure mitigation strategies might be considered. However, we recommend further research to investigate the impact aflatoxin has on growth and development of children as well as exploring the magnitude of aflatoxin exposure with long-time exposure biomarker like AFB1-albumin adduct with a better analytical method (like LC-MS/MS).

## Data Availability

The datasets used and/or analysed during the current study are not publicly available due to limitations of ethical approval involving the patient data and anonymity, but are available from the corresponding author on reasonable request.

## Abbreviations

AFM1: Aflatoxin M1
AFB1: Aflatoxin B1
ELISA: Enzyme-Linked Immunosorbent Assay
EPHI: Ethiopian Public Health Institute
FFQ: Food frequency questionnaire
HDSS: Health and Demographic Surveillance Site
HPLC: High-performance liquid chromatography
ILRI: International Livestock Research Institute
IREC: Institutional Research Ethics Committee
LC–MS/MS: Liquid chromatography-mass spectrometry
OD’s: Optical densities
PACA: Partnership for Aflatoxin Control in Africa
PBS: Phosphate Buffer Saline
PCA: Principal Component Analysis
SES: Socio-economic status

## References

[1] Kensler TW, Roebuck BD, Wogan GN, Groopman JD (2011). Aflatoxin: A 50-year Odyssey of mechanistic and translational toxicology. Toxicol Sci.

[2] Burch DGS, Rowsell C (2001). The Role of Mycotoxins in Pmws – Fact or Fiction. Pig J.

[3] Bankole S, Adebanjo A (2003). Mycotoxins in food in West Africa : current situation and possibilities of controlling it. African J Biotechnol.

[4] Smith LE, Prendergast AJ, Turner PC, Mbuya MNN, Mutasa K, Kembo G (2015). The potential role of mycotoxins as a contributor to stunting in the SHINE Trial. Clin Infect Dis.

[5] Williams JH, Phillips TD, Jolly PE, Stiles JK, Jolly CM, Aggarwal D (2004). Human aflatoxicosis in developing countries: A review of toxicology, exposure, potential health consequences, and interventions. Am J Clin Nutr..

[6] Njumbe Ediage E, Diana Di Mavungu J, Song S, Wu A, Van Peteghem C, De Saeger S (2012). A direct assessment of mycotoxin biomarkers in human urine samples by liquid chromatography tandem mass spectrometry. Anal Chim Acta..

[7] Ezekiel CN, Warth B, Ogara IM, Abia WA, Ezekiel VC, Atehnkeng J (2014). Mycotoxin exposure in rural residents in northern Nigeria: A pilot study using multi-urinary biomarkers. Environ Int.

[8] Wild CP, Turner PC (2002). The toxicology of aflatoxins as a basis for public health decisions. Mutagenesis.

[9] Ayelign A, Woldegiorgis AZ, Adish A, De Boevre M, Heyndrickx E, De Saeger S (2017). Assessment of aflatoxin exposure among young children in Ethiopia using urinary biomarkers. Food Addit Contam Part A Chem Anal Control Expo Risk Assess.

[10] Aflatoxin Impacts and Potential Solutions in Agriculture, Trade, and Health 2014 Available from: https://www.un.org/esa/ffd/ffd3/wp-content/uploads/sites/2/2015/10/PACA_aflatoxin-impacts-paper1.pdf

[11] Ethiopia Demographic and Health Survey 2016 2016 Available from: https://dhsprogram.com/pubs/pdf/FR328/FR328.pdf

[12] Bennett JW, Klich M (2003). Mycotoxins. Clin Microbiol Rev.

[13] Wolde M (2017). Effects of aflatoxin contamination of grains in Ethiopia. Int J Agric Sci.

[14] Alemu T, Berhanu G, Azerefgne F, Skinnes H (2008). Evidence for mycotoxin contamination of maize in Southern Ethiopia: the need for further multidisciplinary research 3rd Int Symp.

[15] Amare A (2010). Mycotoxins and surface and internal fungi of maize from Ethiopia..

[16] USAID; DANYA (2012). Aflatoxin: A synthesis of the research in health, agriculture, and trade.

[17] Butajira HDSS, Ethiopia 2013. Available from: http://indepth-network.org/Profiles/butajira_hdss_2013.pdf

[18] Dewana Z, Fikadu T, Facha W, Mekonnen N (2017). Prevalence and Predictors of Stunting among Children of Age between 24 to 59 Months in Butajira Town and Surrounding District, Gurage Zone. Southern Ethiopia Heal Sci J.

[19] Quantitative Assay for – Aflatoxin M1 in Urine: Helica Biosystems Inc.Cat.No.991AFLM01U-96. 2019. Available from: https://www.hygiena.com/wp-content/uploads/2021/02/Helica-Aflatoxin-M1-Urine-ELISA-Kit-Insert.pdf

[20] Austin PC, Tu JV (2004). Automated variable selection methods for logistic regression produced unstable models for predicting acute myocardial infarction mortality. J Clin Epidemiol.

[21] Njumbe Ediage E, Diana Di Mavungu J, Song S, Sioen I (2013). Desaeger S Multimycotoxin analysis in urines to assess infant exposure: a case study in Cameroon. Environ Int.

[22] Turner NW, Bramhmbhatt H, Szabo-Vezse M, Poma A, Coker R, Piletsky SA (2015). Analytical methods for determination of mycotoxins: An update (2009–2014). Anal Chim Acta.

[23] GIEWS - Global Information and Early Warning System. Food and Agricultural Organization (FAO) of the United Nations. 2019. Available from: https://www.fao.org/giews/countrybrief/country.jsp?code=ETH.

[24] Udoh JM, Cardwell KF, Ikotun T (2000). Storage structures and aflatoxin content of maize in five agroecological zones of Nigeria. J Stored Prod Res.

[25] Chen G, Gong YY, Kimanya ME, Shirima CP, Routledge MN (2017). Comparison of urinary aflatoxin M1 and aflatoxin albumin adducts as biomarkers for assessing aflatoxin exposure in Tanzanian children. Biomarkers..

[26] Ouko E. Sources and levels of human exposure to aflatoxins in Makueni country, KENYA. 2014. Available from: https://www.semanticscholar.org/paper/Sources-and-levels-of-human-exposure-to-aflatoxins-Ouko/6c278e2427f5ad4190649336ebe4d6062778de49

[27] Lei Y, Fang L, Akash MSH, Rehman K, Liu Z, Shi W (2013). Estimation of urinary concentration of aflatoxin M1 in Chinese pregnant women. J Food Sci.

[28] Obuseh FA, Jolly PE, Jiang Y, Shuaib FMB, Waterbor J, Ellis WO (2007). Aflatoxin B1 albumin adducts in plasma and aflatoxin M1 in urine are associated with plasma concentrations of vitamins A and E. J Natl Res Counc Thail Soc Sci..

[29] Kumi J, Dotse E, Asare GA, Ankrah N-A (2015). Urinary Aflatoxin M1 Exposure in Ghanaian Children Weaned on Locally. African J Sci Res..

[30] Ministry of Agriculture: Addis Ababa. AGRO-ECOLOGICAL ZONES OF ETHIOPIA. 2018. Available from: http://hdl.handle.net/123456789/2517

[31] Sumner PE, Lee D. Reducing Aflatoxin in corn during harvest and storage. UGA Coop Ext Bull 1231. 2017;6.

[32] Chen C, Mitchell NJ, Gratz J, Houpt ER, Gong Y, Egner PA (2018). Exposure to aflatoxin and fumonisin in children at risk for growth impairment in rural Tanzania. Environ Int.

[33] Ezekiel CN, Oyeyemi OT, Oyedele OA, Ayeni KI, Oyeyemi IT, Nabofa W (2018). Urinary aflatoxin exposure monitoring in rural and semi-urban populations in Ogun state, Nigeria. Food Addit Contam Part A Chem Anal Control Expo Risk Assess [Internet]..

[34] Yard EE, Daniel JH, Lewis LS, Rybak ME, Paliakov EM, Kim AA (2013). Human aflatoxin exposure in Kenya, 2007: a cross-sectional study. Food Addit Contam Part A Chem Anal Control Expo Risk Assess.

[35] Shuaib FM, Jolly PE, Ehiri JE, Ellis WO, Yatich NJ, Funkhouser E (2012). Socio-demographic determinants of aflatoxin B1-lysine adduct levels among pregnant women in Kumasi. Ghana Ghana Med J.

[36] Leroy JL, Wang JS, Jones K (2015). Serum aflatoxin B_1_ -lysine adduct level in adult women from Eastern Province in Kenya depends on household socio-economic status: a cross sectional study. Soc Sci Med.

[37] Gong YY, Egal S, Hounsa A, Turner PC, Hall AJ, Cardwell KF (2003). Determinants of aflatoxin exposure in young children from Benin and Togo, West Africa: The critical role of weaning. Int J Epidemiol.

[38] Jolly PE, Akinyemiju TF, Jha M, Aban I, Gonzalez-Falero A, Joseph D (2015). Temporal variation and association of aflatoxin B1 albumin-adduct levels with socio-economic and food consumption factors in HIV positive adults. Toxins (Basel).

